# Modeling the dynamics of antibody–target binding in living tumors

**DOI:** 10.1038/s41598-020-73711-y

**Published:** 2020-10-07

**Authors:** Yu Tang, Yanguang Cao

**Affiliations:** 1 Division of Pharmacotherapy and Experimental Therapeutics, UNC Eshelman School of Pharmacy, University of North Carolina, Chapel Hill, NC 27599 USA; 2 Lineberger Comprehensive Cancer Center, School of Medicine, University of North Carolina, Chapel Hill, NC 27599 USA

**Keywords:** Biologics, Drug delivery, Pharmaceutics, Pharmacology

## Abstract

Antibodies have become an attractive class of therapeutic agents for solid tumors, mainly because of their high target selectivity and affinity. The target binding properties of antibodies are critical for their efficacy and toxicity. Our lab has developed a bioluminescence resonance energy transfer (BRET) imaging approach that directly supports the measurement of the binding dynamics between antibodies and their targets in the native tumor environment. In the present study, we have developed a spatially resolved computational model analyzing the longitudinal BRET imaging data of antibody–target binding and exploring the mechanisms of biphasic binding dynamics between a model antibody cetuximab and its target, the epidermal growth factor receptor (EGFR). The model suggested that cetuximab is bound differently to EGFR in the stroma-rich area than in stroma-poor regions, which was confirmed by immunofluorescence staining. Compared to the binding in vitro, cetuximab bound to EGFR to a “slower-but-tighter” degree in the living tumors. These findings have provided spatially resolved characterizations of antibody–target binding in living tumors and have yielded many mechanistic insights into the factors that affect antibody interactions with its targets and treatment efficacy.

## Introduction

The therapeutic antibody is an important class of therapeutics for treating solid tumors. More than 30 therapeutic antibodies have been approved for treating tumors at various stages^1,2^. These broad applications of therapeutic antibodies in solid tumors are largely due to their high target binding selectivity and affinity compared with traditional cytotoxic agents. Once bound to their targets, therapeutic antibodies eradicate tumor cells mainly by three mechanisms: blocking the pathogenic ligand–receptor interactions, triggering cell apoptosis pathways, or activating host effector functions^3^. The mechanisms of action are not exclusive but usually differ depending on the design of the different classes of antibodies.

Regardless of the mode of antibody action, antibody–target engagement is the first and most critical step for antibody efficacy. The patterns of target binding are often associated with the cellular response of the target cells and treatment efficacy. Many studies have revealed that tumor cells can receive information by altering the temporal behavior (dynamics) of their signaling molecules^4,5^. A classic example of this behavior is the extracellular signal-regulated kinase pathway for the epidermal growth factor receptor (EGFR). Transient activation (or blocking) of EGFR is associated with tumor cell proliferation, whereas sustained activation can lead to cell differentiation^6^. In addition, once antibodies have bound to their target cells, they can direct effector cells to elicit antibody-dependent cellular cytotoxicity (ADCC). Thus, the residence time of the antibody–target complex on tumor cells (determined by the off-rate) becomes critical for increasing lipid raft formation and the probability of ADCC^7,8^. Many tumor cells can initiate fast endocytosis upon antibody binding, leading to resistance to antibody attack^9–11^. Therefore, different target binding patterns can lead to distinct cellular reactions and treatment responses.

The target binding affinity is often assessed in vitro, using either surface plasmon resonance (SPR) or ligand competition assays. In SPR analysis, the antibody binds to target molecules that are immobilized on the sensing layer. Binding leads to changes in conformation and the angle of reflectivity, from which the association (k_on_) and dissociation (k_off_) can be quantified^12^. As in other routinely applied technologies that measure binding dynamics, the k_on_ and k_off_ that are determined by SPR merely reflect antibody–target interactions at the molecular level. These techniques are valuable for antibody screening, but they are not relevant to binding under physiological conditions. The target binding properties in living systems remain largely uncharacterized.

Tumor tissues are known to be very heterogeneous, both between and within tumors. In addition to complex tumor genotypes, morphological and phenotypic features can differ, even within the same tumor. The stromal environment where each tumor cell resides largely shapes its phenotypic properties^13^. However, how these stromal components can influence the binding dynamics between an antibody and its targets remains largely undefined. Unlike in vitro assay systems, where all targets are freely accessible, tumors present many physical barriers that influence the diffusion of antibodies, as well as their interactions with the targets^14^. Previous studies have shown that antibodies are unable to freely reach their targets or cannot drift away after dissociating from the targets in the presence of spatial obstacles^15^. The resulting shifts in binding dynamics within living tumors can reduce the cellular response or even lead to treatment failure.

We have developed a bioluminescence resonance energy transfer (BRET) imaging system that can directly monitor the antibody–target binding dynamics in living systems^16^. This imaging system leverages a high signal-to-noise ratio and stringent energy donor–acceptor distance to provide specific measurements of antibody–target binding dynamics in a selective and temporal fashion. It is a minimally invasive system, enabling longitudinal monitoring of in vivo antibody–target interactions. We have previously used this approach to demonstrate that cetuximab binds to its target, EGFR, in a biphasic and dose-shifted manner. In the present study, we have developed spatially resolved computational models for the analysis of the longitudinal imaging data of antibody–target binding in living tumors, and we have compared their binding dynamics in spatially distinct tumor areas. With these models, we have assessed possible mechanisms that could explain the biphasic features of cetuximab–EGFR binding in a xenograft tumor. The results of this study have provided many insights into the dynamic features of antibody–target binding in living tumors and the stroma factors that potentially influence those dynamics.

## Methods

### Study design

**Figure 1:**
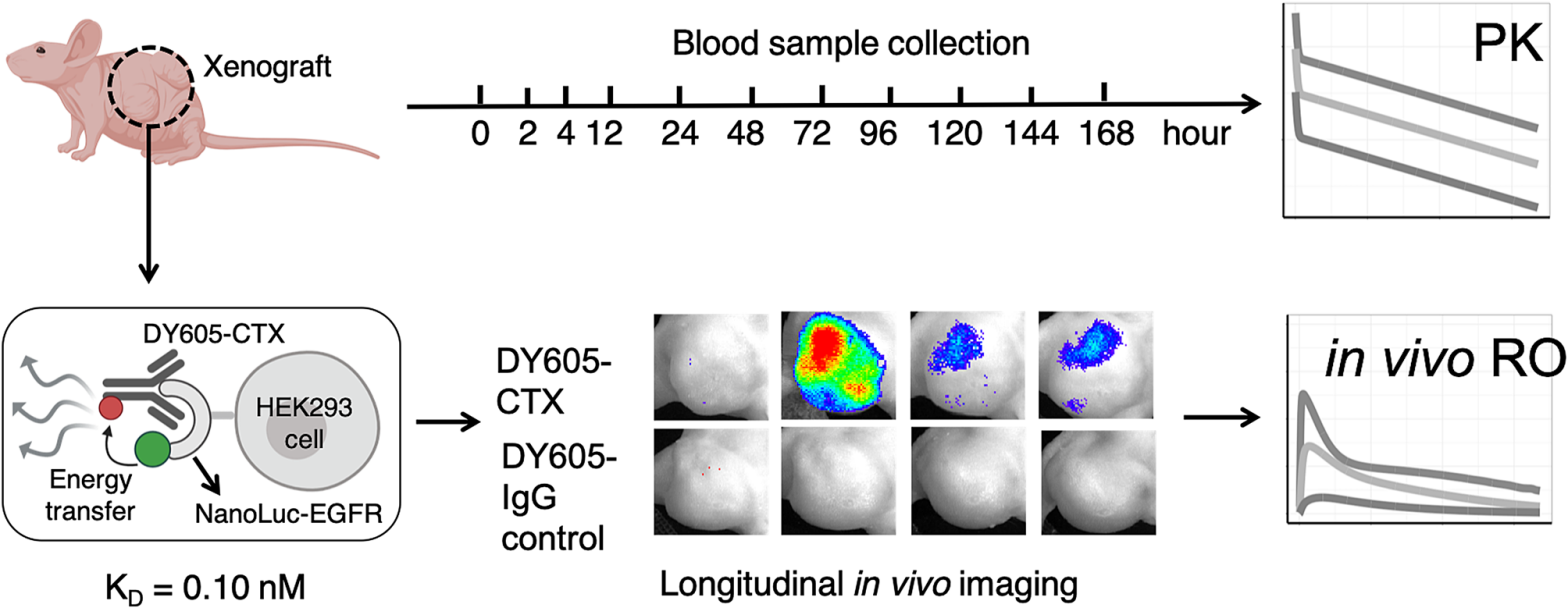
The scheme of the experimental design of the Bioluminescence Resonance Energy Transfer (BRET) study. A small, but bright, luciferase, NanoLuc, was fused to the extracellular domain of EGFR to serve as the energy donor in the BRET pair (Nluc-EGFR). The anti-EGFR antibody cetuximab was labeled with DY605 as the energy acceptor (DY605-CTX). The binding affinity (K_D_) between NLuc-EGFR and DY605-CTX was 0.10 nM. Twenty nude mice were inoculated with NLuc-EGFR-expressing HEK293 cells; and the BRET imaging study was performed after xenograft tumor sizes had reached 500 mm^3^. DY605-CTX at three doses (1.0, 8.5, and 50 mg/kg) or DY605-human IgG was injected via tail vein (n = 5/dose group). Blood samples (30 µL) were collected at designated times for pharmacokinetics assessment. The plasma concentrations of DY605-CTX were quantified based on fluorescence intensities. Images were acquired using an IVIS Kinetic optical imaging system upon administration of the NanoLuc substrate furimazine (i.v., 0.25 mg/kg). The fluorescence intensity was determined to quantify the concentrations of the antibody–target complex and to derive the receptor occupancy (RO). The tumors were collected at the end of the study (around 192 h) and snap-frozen in liquid nitrogen.

Our lab has developed a BRET approach to support the investigations of antibody–target binding dynamics in the native tumor environment. Specifically, a small but bright luciferase, NanoLuc, was fused to the extracellular domain of EGFR to serve as the energy donor in the BRET pair^16^. An anti-EGFR antibody, cetuximab, was labeled with DY605, a fluorophore with an emission wavelength at 625 nm, to serve as the energy acceptor. Prior to the binding between DY605-labeled cetuximab (DY605-CTX) and the NanoLuc-fused EGFR (NLuc-EGFR), the distance of NanoLuc to the DY605 was too large to trigger BRET, and only the bioluminescence emission at 460 nm for NanoLuc was observed. However, binding of DY605-CTX to NLuc-EGFR increased the proximity between NanoLuc and DY605 and allowed the transfer of bioluminescence energy to DY605 and the emission of fluorescence signals at 625 nm (Fig. 1). The binding affinity between DY605-CTX and NanoLuc-EGFR was 0.10 nM, which is in agreement with previously reported K_D_ for CTX-EGFR binding (0.20 nM) (Fig. 1)^17^. The k_on_ of DY605-CTX: NanoLuc-EGFR binding was about 0.20 nM^−1^ min^−1^ and the k_off_ was 0.02 min^−1^ (Fig. S1), which are close to the constants of CTX-EGFR binding measured by SPR^18^. The DY605-CTX: NanoLuc-EGFR BRET pair allowed robust and reliable quantification of CTX-EGFR interaction.

The experimental design is shown in Fig. 1. In total, 20 nude mice were inoculated with NLuc-EGFR-expressing HEK293 cells. To establish tumor models, 5 × 10^6^ NLuc-EGFR HEK293 cells were suspended in 0.1 mL of PBS/ Matrigel (1/1, v/v) and inoculated subcutaneously into the inguinal flank of the nude mice. The BRET imaging study was performed when the tumor sizes had reached 500 mm^3^. The imaging study was initiated by injecting the DY605-labeled cetuximab via the tail vein of the xenograft mice at three doses: 1.0, 8.5, and 50 mg/kg (n = 5 /dose group), or DY605-labeled human IgG (n = 5). Blood samples (30 µL) were collected at the designated times for pharmacokinetics (PK) assessment. The plasma concentrations of cetuximab were quantified based on fluorescent intensities. Images at both 460 nm and 625 nm were acquired using an IVIS Kinetic optical imaging system (Caliper Life Sciences, Alameda, CA, USA) upon administration of NanoLuc substrate furimazine (i.v., 0.25 mg/kg). The fluorescence intensity was determined to quantify the concentrations of the antibody–target complex, and the receptor occupancy (RO) was calculated (Supplementary methods). The tumors were collected at the end of the study (at approximately 192 h) and snap-frozen in liquid nitrogen.

### Plasma PK model

**Figure 2:**
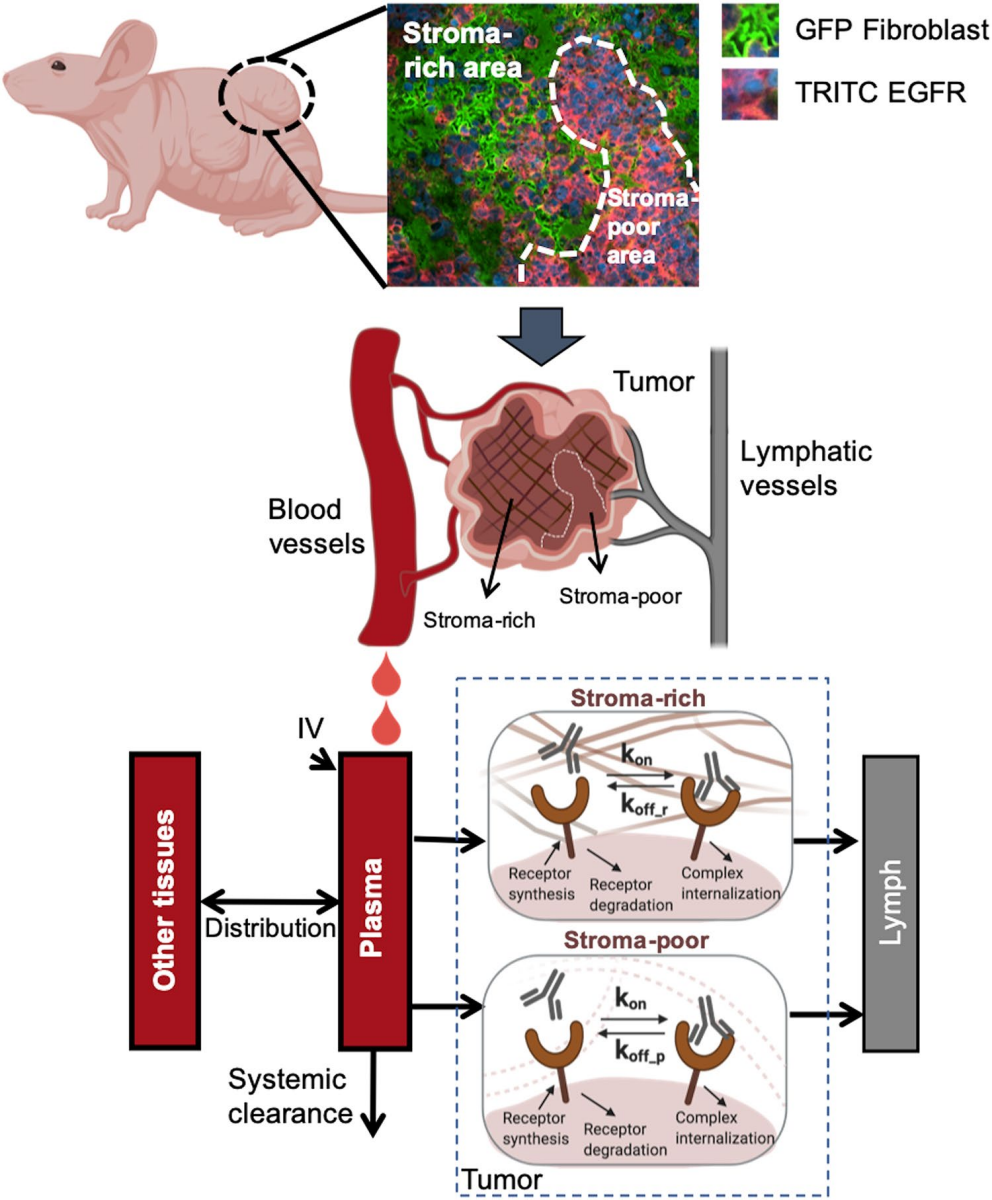
The spatially resolved computational model describing the antibody–antigen binding kinetics in xenografts. The antibody plasma pharmacokinetics were described using a two-compartment model with a linear tissue distribution and a linear systemic clearance. The solid tumors were conceptually dissected into two anatomical compartments—stroma-rich and stroma-poor areas—to account for the spatial heterogeneity, as seen in the staining slide. The stroma-poor compartment described the area where tumor cells grow without any spatial restriction by stromal cells, whereas the stroma-rich tumor compartment represented the area where tumor cells grow in the presence of dense tumor-associated stromal cells (e.g., fibroblasts). Antibodies were assumed to extravasate from tumor blood vessels into the interstitial space and leave the interstitial space via lymphatic vessels. In both tumor compartments, the free receptors were synthesized and degraded on the tumor cells. The antibody–receptor complexes were cleared by internalization. The free antibodies bound to free receptors at a rate of k_on._ The antibodies dissociated from receptors at a rate of k_off_r_ in the stroma-rich compartment and a rate of k_off_p_ in the stroma-poor compartment.

The antibody plasma PK was described using a two-compartment model with a linear tissue distribution (CL_D_) and a linear systemic clearance (CL_P_) (Fig. 2). The PK data in three dose groups (1.0, 8.5, and 50 mg/kg) were analyzed simultaneously using the PK model, using a naïve pooled-data (NPD) approach. The volume of plasma (V_plasma_) was set to 0.001 L for 20 g mice^19^.

### Modeling antibody–target binding dynamics in tumors

The dynamics of antibody–target binding in solid tumors were further characterized to obtain mechanistic insights by implementing a sequential modeling strategy. Here, the PK model was first optimized and then fixed during the second step to explore the antibody–target binding dynamics.

The solid tumors were conceptually dissected into two anatomical compartments: a stroma-rich and a stroma-poor area, to account for the spatial histological heterogeneity (Fig. 2). The stromal-rich compartment described the area where tumor cells grew relatively quickly, without any spatial restriction by stromal cells. By contrast, the stroma-poor tumor compartment represented the area where tumor cells grew in the presence of dense tumor-associated stromal cells (e.g., fibroblasts). The relative volume and blood flow in the two tumor areas were evaluated as model parameters.

The extravasation of the antibody from the tumor blood vessels to the interstitial space was assumed to be dominated by convection and was described by a vascular reflection coefficient (${\upsigma }_{\mathrm{v}})$ and the convective lymph flow into either the stroma-rich area (L_r_) or the stroma-poor area (L_p_). The value of ${\upsigma }_{\mathrm{v}}$ was set at 0.78, a value reported for subcutaneous xenograft models^19^. The values of L_p_ and L_r_ were functions of the tumor blood flow (TBF)^20^, the total tumor volume (V_tumor_), and the relative fraction between the two tumor areas (f_t_), as described in the following equations:

$${\mathrm{L}}_{\mathrm{r}}={((1-\mathrm{f}}_{\mathrm{t}})\cdot {{\mathrm{V}}_{\mathrm{tumor}}\cdot \mathrm{TBF}}_{\mathrm{r}})\cdot {\mathrm{f}}_{\mathrm{L}}$$

$${\mathrm{L}}_{\mathrm{p}}={(\mathrm{f}}_{\mathrm{t}}\cdot {{\mathrm{V}}_{\mathrm{tumor}}\cdot \mathrm{TBF}}_{\mathrm{p}})\cdot {\mathrm{f}}_{\mathrm{L}}$$

where TBF_p_ and TBF_r_ describe the tumor blood flows in two tumor areas. The value of f_L_ was set to 0.2%^21^. The total tumor volumes (V_tumor_) were measured using a caliper.

The spaces for antibody distribution and for antibody–target interaction in both tumor compartments were set to a fraction (f_av_) of the total interstitial space.

$${\mathrm{V}}_{\mathrm{r}\_\mathrm{a}}={(1-\mathrm{f}}_{\mathrm{t}})\cdot {{\mathrm{V}}_{\mathrm{tumor}}\cdot \mathrm{f}}_{\mathrm{isf}}\cdot {\mathrm{f}}_{\mathrm{av}}$$

$${\mathrm{V}}_{\mathrm{p}\_\mathrm{a}}={\mathrm{f}}_{\mathrm{t}}\cdot {{\mathrm{V}}_{\mathrm{tumor}}\cdot \mathrm{f}}_{\mathrm{isf}}\cdot {\mathrm{f}}_{\mathrm{av}}$$

The available fraction f_av_ was set at 27.5%^19,22^. Notably, the antibody–antigen bindings occurred in the same space that was accessible to antibodies (V_r_a_ and V_p_a_). The target (i.e., EGFR) was assumed to be synthesized by tumor cells at a zero-order rate constant (k_syn_) and to be endocytosed at a first-order rate constant (k_deg_). The cetuximab-EGFR complex was assumed to be internalized by the tumor cells at a first-order rate constant (k_int_). All parameters regarding the target protein (k_syn_, k_deg_, and k_int_) were assumed to be conserved inside the tumors. The association rate between the antibody and target is denoted as k_on_ and the dissociation rate constant is k_off_.

We used the developed modeling framework primarily to investigate two competing hypotheses: (1) the antibodies bind to the targets differentially across two tumor areas (the heterogeneous binding model, HBM) and (2) the antibodies are distributed differentially into two tumor areas, but with the same binding profile (the heterogeneous distribution model, HDM). The differential equations for both models are provided in the Supplementary methods. We optimized both models against the data and evaluated which model made more consistent predictions to the observed RO data. The selection of the most suitable model and the parameter estimates were confirmed by visual inspection, the measured data vs. individual conditional model prediction plot, the individual conditional standardized residual vs. model prediction plot, the individual conditional standardized residual vs. time plot, the CV% of estimated parameters, and the physiological plausibility of the estimated parameters.

### Immunofluorescence (IF) staining

We assessed the spatial distributions of the antibody in tumors by IF staining after the imaging study. The tumor samples were preserved and sliced in OCT medium (Fisher scientific, Waltham, MA, USA). The sliced tumor tissues were fixed in methanol/acetone (1:1) at 4 °C. After blocking with phosphate buffered saline (PBS) containing 2% fetal bovine serum (FBS) (Millipore Sigma, Burlington, MA, USA), the tumor slices were stained with anti-EGFR and anti-α-SMA antibodies. In brief, the slides were incubated with Alexa Fluor 555-conjugated primary rabbit anti-human EGFR antibodies (Thermo, Waltham, MA, USA, 1:100 diluted in PBS) and primary mouse anti-mouse α-SMA antibodies (Thermo, Waltham, MA, USA, 1:500 diluted in PBS) at 4 °C overnight and then incubated with Alexa Fluor 488-conjugated goat anti-mouse antibodies (Thermo, Waltham, MA, USA, 1:2000 diluted in PBS) at room temperature for 1 h. The immunofluorescence images were acquired with a Live Cell Imaging Microscope (Nikon, Melville, NY, USA).

### Model simulation

The developed model was applied to simulate the profiles of antibody–target binding dynamics and the resultant RO at different conditions. The concentrations of free antibodies, free targets, and antibody–target complexes in both tumor areas were simulated and compared. In addition, the SPR-measured binding parameters were applied to replace the optimized parameters to allow an examination of differences in antibody–EGFR binding dynamics in the living tumors versus in vitro binding in buffers.

### Ethical statement

All animal procedures were carried out in accordance with the Guide for the Care and Use of Laboratory Animals as adopted and promulgated by the US National Institutes of Health, and were approved by the Institutional Animal Care and Use Committee (University of North Carolina, Chapel Hill, NC).

## Results

### Plasma PK and antibody–target binding dynamics in tumors

In this study, DY605-CTX showed bi-exponential and linear PK profiles^16^, as the area under the curve (AUC) and the peak plasma concentrations increased proportionally to the doses. A temporal shift was observed from the antibody plasma PK to the ROs in the tumors. The tumor ROs peaked at approximately 4 h post-dosing, which was consistent across doses, suggesting that the extravasation of DY605-CTX into tumors is a slow and linear process. The increase in the tumor ROs was less than dose proportional, indicating a nonlinear process was involved in the conversion of free antibodies in the plasma to bound antibodies in tumors. Notably, the target EGFR in the tumors was not saturated, even at a supra-therapeutic antibody dose (50 mg/kg), suggesting fractional target accessibility. Furthermore, the RO profiles declined in a biphasic manner and showed a shallow terminal declining phase, particularly at the two higher doses. Interestingly, the transition from the rapid to the slowly declining phases was not consistent across doses.

### The antibody–target binding profiles in tumors were well recapitulated by the HBM

**Figure 3:**
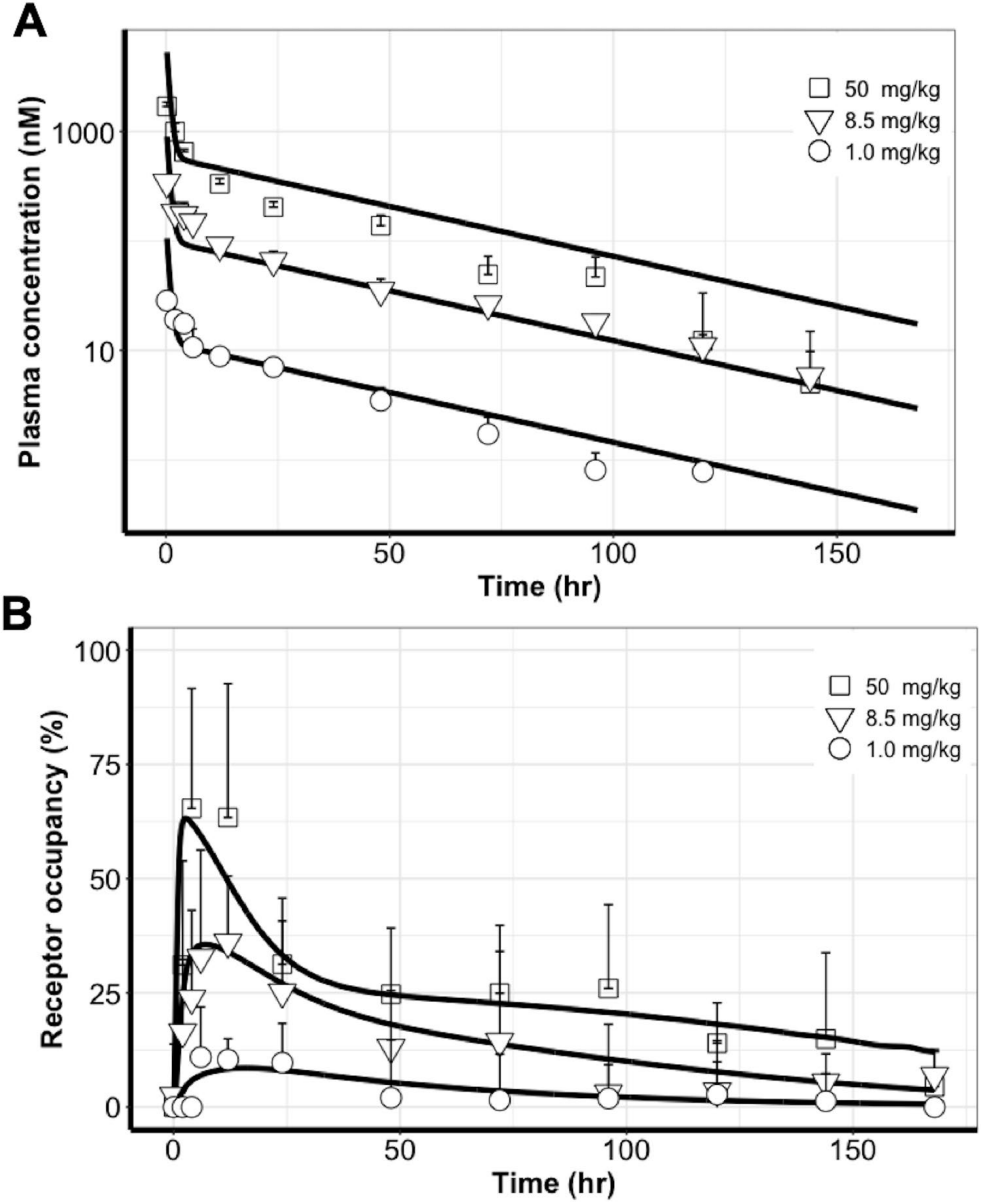
The PK profiles were well captured by a two-compartment model and the profiles of antibody–target binding in tumors were well recapitulated by the heterogeneous binding model (HBM). (**A**) The two-compartment PK model adequately recapitulated the PK profiles at all doses. (**B**) The tumor receptor occupancy (RO) profiles were well characterized by the heterogeneous binding model (HBM) at three doses. Each data point represents the mean plasma concentration or mean RO. Error bars represent $\pm$ SD.

The average plasma concentrations and tumor ROs were used for model competition. As shown in Fig. 3A, the two-compartment PK model adequately recapitulated the PK profiles at all doses. This confirms the linear PK properties of DY605-CTX in xenograft mice within the assessed dose range. The estimated PK parameters and CV% are shown in Table 1. The estimated systemic clearance and volume of tissue distribution were consistent with those of previous reports.

**Table 1:** Pharmacokinetics parameter estimations.

Parameter	Unit	Definition	Estimation (CV%)
CL_D_	L$\cdot$h^−1^	Tissue distribution flow	0.0012 (15%)
CL_p_	L$\cdot$h^−1^	Cetuximab systemic clearance	0.00021 (4.8%)
V_other_	L	Other tissue volume	0.0076 (7.1%)
V_plasma_	L	Plasma volume	0.001 (fixed)

**Figure 4:**
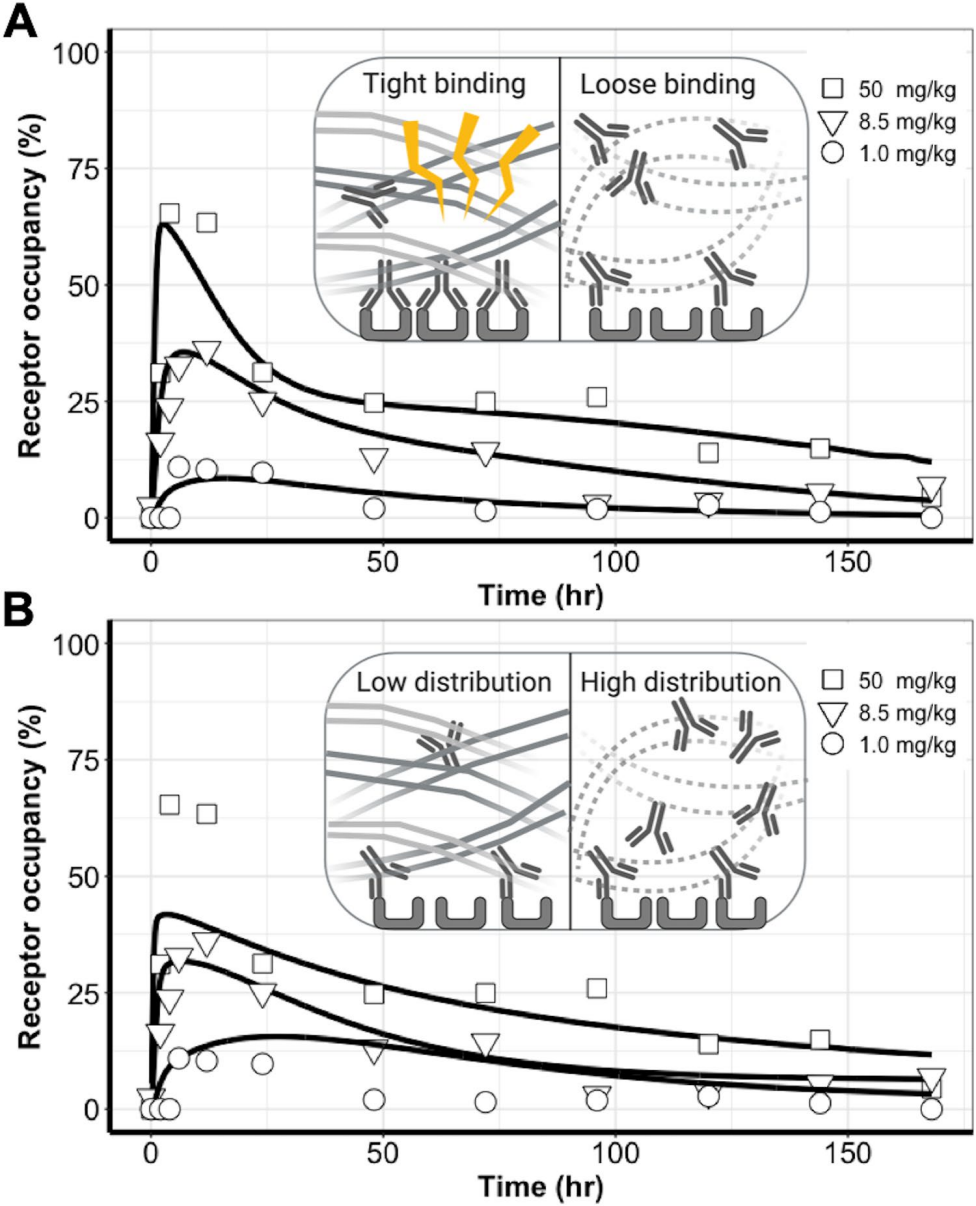
Uneven distributions of antibodies in tumors could not sufficiently explain the observed binding dynamic features in comparison to the heterogeneous binding patterns. (**A**) The heterogeneous binding model (HBM) well-captured the antibody–target binding kinetics, whereas (**B**) The heterogeneous distribution model (HDM) failed to capture the receptor occupancy (RO) profiles across three doses, and a clear model misspecification was indicated.

The tumor RO profiles were well characterized by the HBM at all three doses (Figs. 3B and 4A). The model suggested different antibody–target binding dynamics across two tumor spatial areas. The optimized parameters are shown in Table 2. The association rates (i.e., k_on_) between cetuximab and its target EGFR in both tumor compartments were estimated to be close and were therefore considered as a shared parameter in the two tumor compartments. The estimate of k_on_ was 0.03 nM^−1^$\cdot$h^−1^, a value approximately 1% of the rate measured using SPR^18^, indicating the impact of physical barriers on the association rate in the living tumors compared to the in vitro buffer conditions. Interestingly, the complex dissociation rate (i.e., k_off_p_ and k_off_r_) was markedly different between the two tumor areas. The optimized k_off_p_ was 0.61 h^−1^ in the stroma-poor tumor area, which is close to the SPR measured values. However, the complex dissociation rate was estimated as much slower (k_off_r_ = 0.0017 h^−1^) in the stroma-rich tumor area^18^. The estimated endocytosis rate (k_int_ in Table 2) revealed the net endocytosis rate of EGFR antigen after subtracting the fraction of recycling, which explains the relatively slower endocytosis rate estimated in our model compared to literature values^23^.

**Table 2:** Heterogeneous binding model (HBM) parameter estimations.

Parameter	Unit	Definition	Estimation (CV%)
k_deg_	h^−1^	EGFR degradation rate	0.013 (42%)
R0	nM	EGFR initial concentration in tumor stroma-rich and stroma-poor space	0.0020 (73%)
k_on_	nM^−1^$\cdot$h^−1^	Cetuximab-EGFR apparent association rate	0.030 (53%)
k_off_p_	h^−1^	Cetuximab-EGFR apparent dissociation rate in stroma-poor regions	0.61 (55%)
k_off_r_	h^−1^	Cetuximab-EGFR apparent dissociation rate in stroma-rich regions	0.0017 (54%)
f_t_	/	Ratio of tumor stroma-poor volume over total space	0.89 (5.8%)
TBF	h^−1^	Tumor blood flow per 1 L tumor	9.2 (21%)
k_int_	h^−1^	Cetuximab-EGFR internalization rate	0.14 (26%)

Notably, HBM elucidated the heterogeneous binding in living tumors in a robust and physiologically-relevant manner. The diagnostic plots showed the goodness of fitting of HBM (Fig. S2A). The final parameters of HBM were physiologically-relative (Table 2). For example, TBF was estimated to be 15.4 mL/100 mL/min and comparable to reported xenograft tumor blood flow (27.5 mL/100 mL/min)^20^. HBM was not sensitive to R0, which cannot be experimentally measured (Fig. S2B).

Unfortunately, as shown in Fig. 4B, the HDM failed to capture the RO profiles across the three doses and a clear model misspecification was indicated. Even with a sharp distribution gradient across tumor areas, the model could not provide a good prediction of the biphasic dynamic feature in the RO profiles. The RO peaks at 50 mg/kg were drastically under-predicted, while the RO values at 1.0 mg/kg were over-predicted. The poor performance of HDM indicated that heterogeneous antibody distribution could not be the primary mechanism for the biphasic declining feature of antibody–target binding in tumors. The parameter estimations of HDM are presented in Table S1.

The different performance between HBM and HDM was also indicated by the Akaike information criterion (1505 vs. 1520). We concluded that the biphasic dynamic feature of RO was better explained by the different binding profiles than by the different distribution profiles in the two tumor compartments. Therefore, we selected the HBM as the model for further exploration.

### Cetuximab persisted longer in the stroma-rich area than in the stroma-poor area

**Figure 5:**
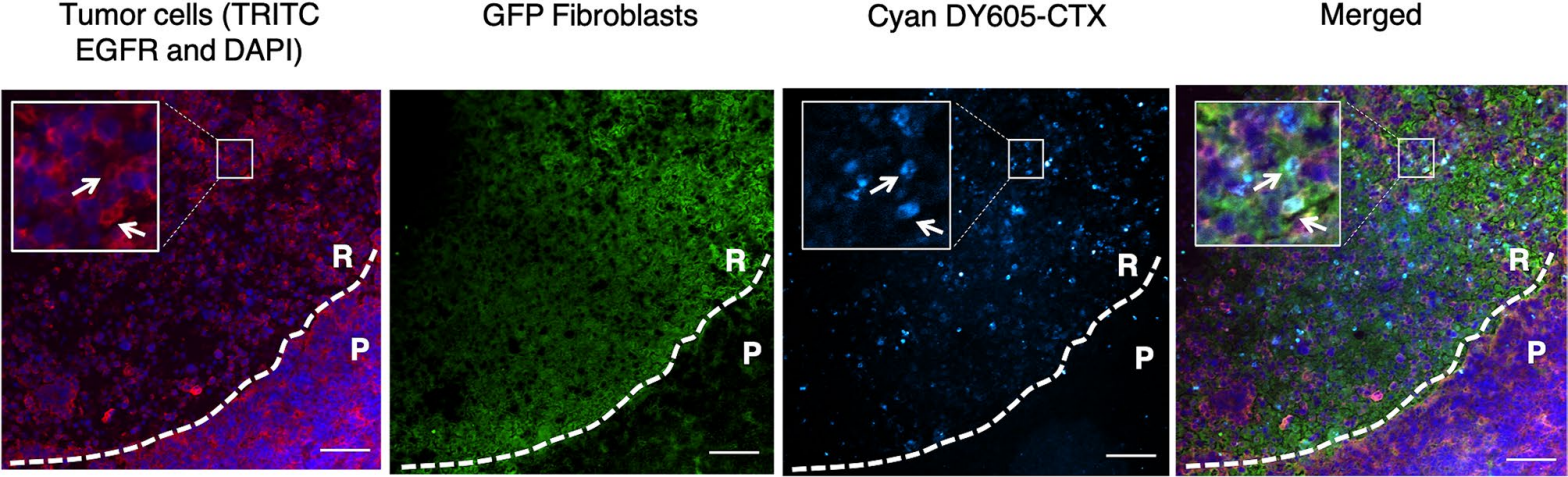
Cetuximab persisted longer in the stroma-rich area than in the stroma-poor area. A representative immunofluorescence (IF) image shows the histology of the tumor collected at the end of the bioluminescence resonance energy transfer (BRET) imaging study, revealing the spatial distribution of the antibody (Cyan DY605-CTX), tumor-associated fibroblast (GFP Fibroblasts), and EGFR-positive tumor cells (TRITC EGFR and DAPI). Area P represents the tumor area without many stroma cells and with evenly distributed tumor cells. Area R represents the stroma-rich area, where tumor cells were surrounded by tumor-associated fibroblasts. Cyan signal denoted the total antibodies, including free DY605-CTX and bound DY605-CTX. As the white arrows indicate, residual antibodies are present at the tumor cell surfaces and largely overlap with EGFR staining, indicating the bound DY605-CTX. Scale bar = 100 $\mathrm{\mu m}$.

At the end of the imaging study, we sectioned the tumors and stained the EGFR-positive tumor cells, tumor-associated fibroblasts, and cetuximab to evaluate the residual antibodies and their spatial distributions. Figure 5 shows representative images of the spatial distribution of the antibody (DY605-CTX), tumor-associated fibroblast (GFP Fibroblasts), and EGFR-positive tumor cells (TRITC EGFR). Area P represents the tumor area without many stroma cells and with evenly distributed tumor cells. Area R represents the stroma-rich area, where tumor cells were surrounded by tumor-associated fibroblasts. As shown in Fig. 5, by 192 h after antibody dosing, some antibodies were still present in the stroma-rich area, while detection of antibodies in the stroma-poor area was negligible. No residual antibodies were observed for nonspecific IgG, suggesting that the residual antibodies were associated with Fab binding and not with non-specific binding (Fig. S3A).

A close inspection of the spatial location indicated that residual antibodies had a very high co-localization with tumor cells (Fig. 5). We observed that most of the residual antibodies in the stroma-rich area were retained on the surfaces of the tumor cells, likely as antibody–target complexes. This observation is consistent with our model predictions whereby antibodies would dissociate from the target much more slowly in the stroma-rich area. Only a small amount of bound antibodies was observed at the edge of Area P, and most antibodies had been degraded in the stroma-poor tumor area. In addition, the total EGFR was sparser in Area R than in Area P, suggesting a higher EGFR suppression in the stroma-rich tumor area. These findings agreed with the model simulations described in the next section. More representative images were shown in Fig. S3B.

### Cetuximab bound EGFR at a “slower-but-tighter” degree in living tumors than in the in vitro conditions

**Figure 6:**
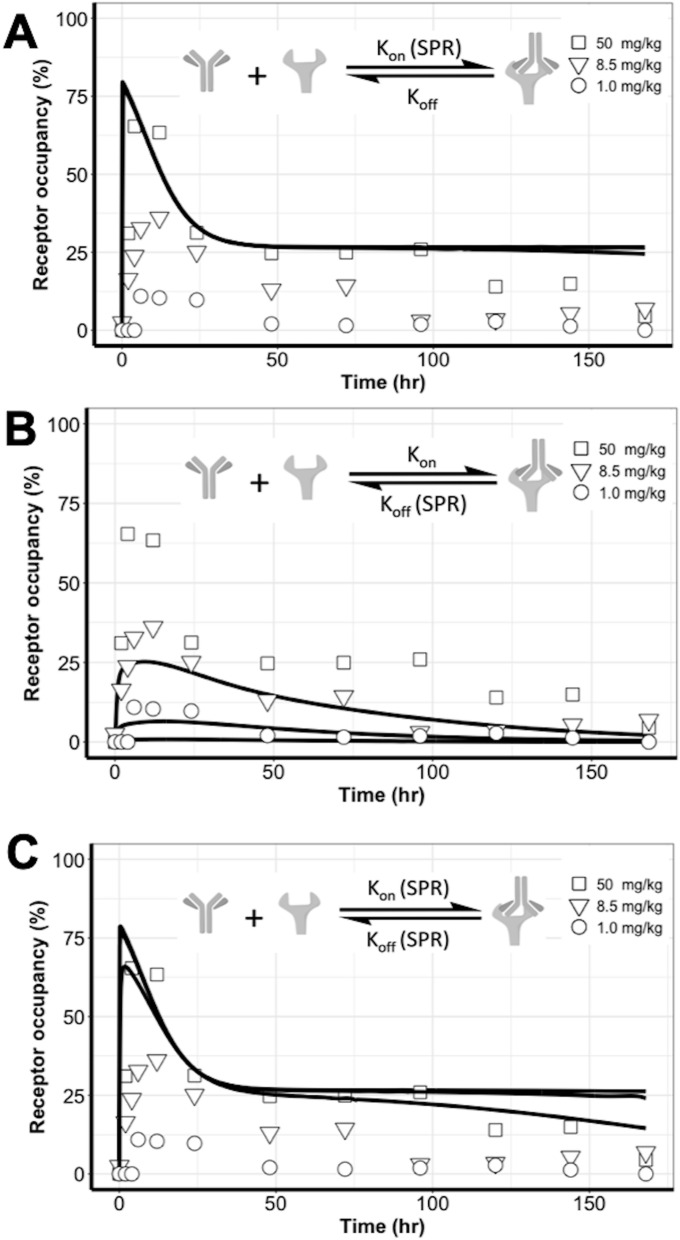
Cetuximab bound EGFR at a “slower-but-tighter” degree in the living tumors than in the in vitro conditions. The target binding parameters in the heterogeneous binding model (HBM) were replaced with the SPR-measured values to predict the receptor occupancy (RO) profiles at three doses; these are superimposed on the experimental observations. (**A**) When k_on_ was set to a SPR-measured value in both tumor compartments, the model over-predicted the RO. No difference was detected in the predicted RO profiles across the three doses. (**B**) When a SPR-measured k_off_ was used in both tumor compartments, the model under-predicted the RO data. (**C**) SPR-measured k_on_ and k_off_ values could not differentiate the RO profiles across the three doses.

We further examined antibody binding dynamics in tumors in comparison to the binding in the in vitro condition, which is usually measured using SPR methods. We replaced the target binding parameters in the HBM with the SPR-measured values to predict the RO profiles at three doses, which were superimposed on the experimental observations. When k_on_ was set to an SPR-measured value in both tumor compartments, the model over-predicted the RO. Even though the biphasic feature on RO curves was predicted, almost no difference was detected in the predicted RO profiles across the three doses (Fig. 6A). With the SPR-measured k_off_ in both tumor compartments, the model under-predicted the RO data (Fig. 6B). The biphasic declining feature disappeared in this parameter setting. When both target binding parameters were set to SPR-derived values, the model also over-predicted ROs (Fig. 6C). Collectively, these findings confirmed a marked difference in antibody–target binding dynamics between the living tumors and the in vitro buffer systems.

### Cetuximab durably suppressed free EGFR but transiently formed antibody–target complexes in living tumors

**Figure 7:**
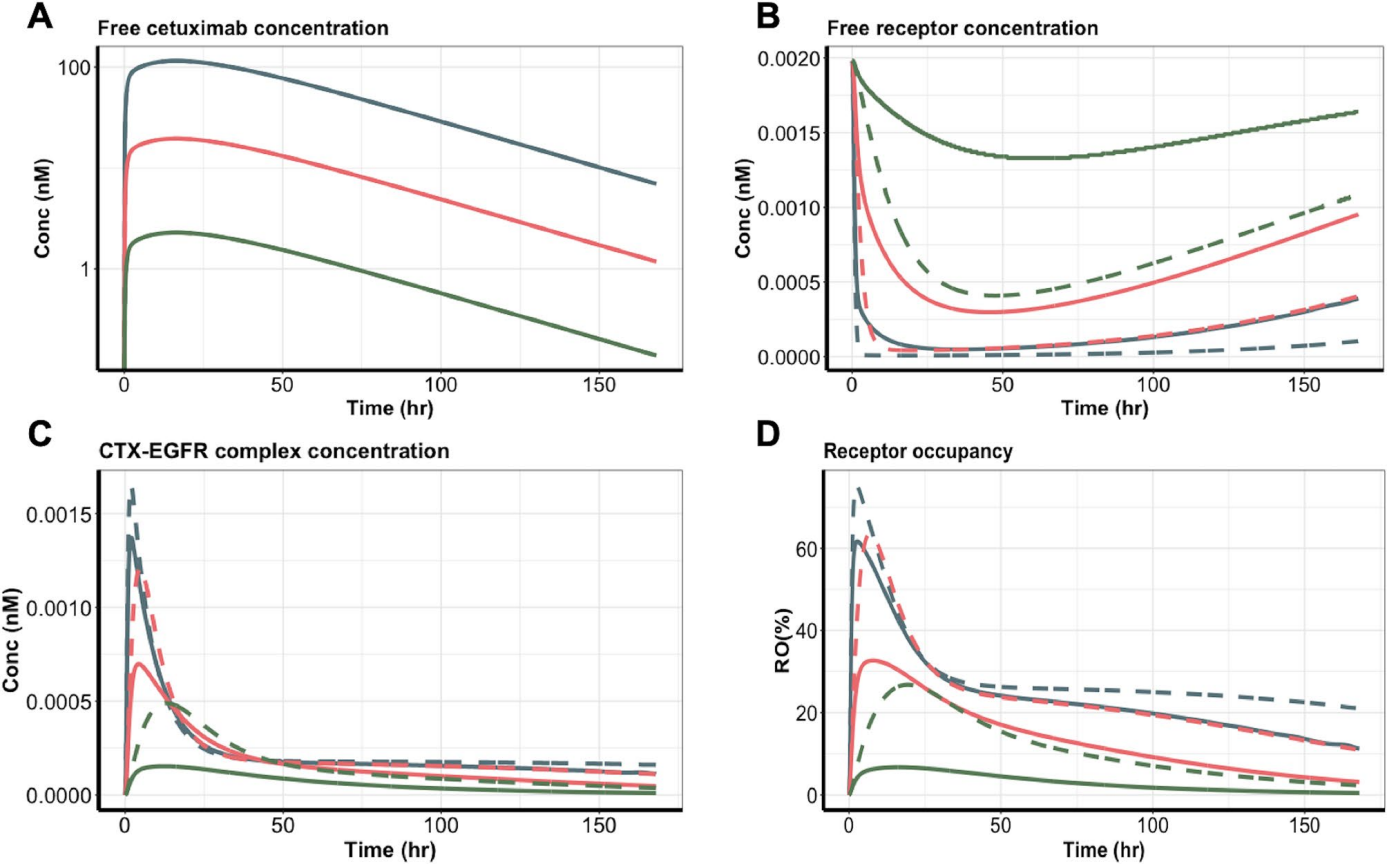
Cetuximab durably suppressed free EGFR, but the complex was formed transiently in tumors. (**A**) The free cetuximab concentration, (**B**) free EGFR concentration, (**C**) cetuximab-EGFR complex concentration, and (**D**) receptor occupancy in the two tumor compartments were simulated based on the heterogeneous binding model (HBM). Blue, red, and green lines represent the three cetuximab dose groups: 50, 8.5, and 1.0 mg/kg. Solid lines represent the tumor stroma-poor compartment, whereas the dashed lines represent the tumor stroma-rich compartment.

We simulated free cetuximab, free EGFR, cetuximab-EGFR complexes, and the RO in both tumor areas. The free cetuximab was similar in both tumor compartments at all doses (Fig. 7A), consistent with the model assumption. Free EGFR was rapidly suppressed, and the suppression lasted for over 150 h, particularly at 50 mg/kg (Fig. 7B). At all doses, the free EGFR in the stroma-rich areas (as indicated by the dash lines) was suppressed to a higher degree, primarily because of a relatively lower fraction of EGFR-positive tumor cells and a tighter antibody-EGFR binding in the stromal-rich tumor areas. Those findings indicated that cetuximab was predicted to have a stronger suppressive effect on free EGFR in the stroma-rich area. The magnitude and duration of EGFR suppression were both dose dependent, so a higher antibody dose gave a larger and longer suppression of the free target (Fig. 7B).

Compared to the durable target suppression, the formation of the antibody–target complex was quite transient (Fig. 7C). In both tumor compartments, the complex concentrations peaked at around 5 h after dosing, but the complex decayed shortly after the peaks and was subsequently maintained at relatively low levels for an extended period. A small difference was evident in complex concentrations across doses during the terminal phase. Despite the similar concentrations in the two tumor areas, the different binding properties of cetuximab meant that it showed relatively higher and more durable RO in the stroma-rich than in the stroma-poor areas (Fig. 7D). The difference between the two tumor areas became smaller as the dose increased.

## Discussion

Our understanding of antibody–target interactions, particularly in the native physiological context, is still limited, mainly due to the lack of approaches to detect their binding dynamics with temporal and spatial resolution or specificity^24^. For example, immunohistochemistry (IHC) staining can quantify the spatial distribution, but it often fails to incorporate the dynamic factors present in physiological situations^25^. Most in vivo imaging methods often cannot distinguish signals arising due to specific target engagement versus nonspecific signals^16^. We previously developed a BRET method that enables longitudinal monitoring of the binding dynamics between the antibody and its target in living tumors^16^. Using this method, we observed biphasic and dose-shifted binding dynamics between cetuximab and its target EGFR. In the present study, we developed a spatially resolved computational model to disentangle the dynamic binding patterns and evaluate the mechanisms.

Heterogeneously-distributed tumor stromal cells are likely to cause uneven spatial restrictions and mechanical stress in the solid tumors, possibly altering antibody-antigen interactions^26^. Our model accounted for this heterogeneity in solid tumors and elucidated the antibody binding dynamics. Compared to the in vitro systems, an antibody in a living tumor could bind to its target to a “slower-and-tighter” degree. We observed that cetuximab was bound differently to its target in the stroma-rich areas than in the stroma-poor regions (Table 2). Cetuximab had a much slower apparent dissociation rate in the stroma-rich areas, which was confirmed by immunofluorescence staining. This finding agreed with the longitudinal tumor staining results reported before, in which the higher bound antibodies in the stroma-rich regions were observed^27–29^. For example, using fluorescently and immunohistochemically stained tumor cryosections, cetuximab and trastuzumab showed higher target-bindings at the stroma-rich area at the 24 h post-administration^27,28^. The binding features in the stroma-rich tumor area were consistent with experimental observations that the stress stroma could restrict the diffusion of antibodies in the tumors^30^. This restricted diffusion could reduce both association and dissociation rates. Another possible reason for the lower dissociation rate in the stroma-rich tumor regions was the intense extracellular matrix, which would prevent the antibody from easily drifting away from the binding zone, thereby resulting in a high fraction of rebinding^15^. The slow dissociation rate resulted in the accumulation of residual antibodies in the stroma-rich tumor areas, even when the systemic antibodies had been largely eliminated.

We developed two spatially resolved computational models by assuming either heterogeneous binding or heterogeneous distribution between the stroma-rich and stroma-poor tumor regions (Fig. 4). We then used competition studies to test which of the two models would consistently predict the observed the dynamic features of cetuximab–EGFR binding. The performance was much better for the model with the heterogeneous binding assumption than with the heterogeneous distribution, indicating that an uneven distribution of antibodies in two tumor areas was not the primary reason for the biphasic declining features in the tumor RO data. One clarification should be made, namely that the inconsistent predictions produced by the HDM only suggest that the uneven distributions of antibodies in tumors do not sufficiently explain the observed binding dynamic features. Therefore, this precludes making the implication of uniform distribution of antibodies in the tumor. Antibody exhibited high perivascular distributions in tumors due to its large size and charges. Of note, our analysis was to compare antibody distributions between two histologically different tumor areas, rather than to evaluate antibody distribution gradients around tumor vessels.

Another interesting finding is that cetuximab durably suppressed free EGFR but transiently formed antibody–target complexes in living tumors (Fig. 7). Cetuximab eradicates tumor cells partially by downregulating the EGFR expression^31^. ADCC was also observed in cetuximab tumor-killing effects, yet the impact of ADCC on cetuximab treatment effects remains unclear. We found that the suppression in EGFR is more sustained than cetuximab-EGFR formation, suggesting that the cetuximab-induced EGFR downregulation could have a more prolonged impact on cetuximab treatment effects compared to ADCC. Further exploration in the antibody-antigen binding dynamics will provide more insights into the antibody mechanism of actions.

One limitation of this study was that the model was developed based on the imaging data in xenografts, which may not recapitulate the complexity of clinical tumors. Compared to the HEK293 derived xenograft, clinical tumors such as pancreatic adenocarcinoma usually consists of high but varied stromal components, limiting antibody accessibility to tumor cells and shifting the binding dynamics. Many drug delivery systems have been recently developed to target tumor-associated stromal cells to improve target accessibility and binding properties^32–35^. Our approach can support in-depth investigations of the stromal effects on antibody–target binding dynamics in various types of tumor microenvironments. Furthermore, two distinctive tumor compartments for stroma-rich and stroma-poor areas are subjective. The tumor vascular structure was not considered in the model, which may influence the model results concerning the varying degrees of vascularization and vessel membrane structures between stroma-rich and stroma-poor tumor areas. The binding kinetic parameters and antibodies strongly influenced the spatial distribution of antibodies within tumor tissues. Antibodies with relative lower K_D_ values showed strong perivascular distribution within tumor tissues, also known as “binding-site barrier” effect, which further influence the anti-tumor effects^36–38^. To fully account for the spatial gradient, the diffusion–reaction equation should be more appropriate, but it had an identifiability issue during model optimization. In addition, the binding parameters we estimated from the models are all apparent values that account for the influence of the physical and stromal factors in the tumor microenvironment. The IF staining images were acquired at the end of the BRET imaging study, which was preferably conducted in a longitudinal manner to match our model simulation.

Overall, in the present study, we combined the strengths of BRET imaging and spatially resolved computational models to evaluate the dynamics of binding an antibody to its target in living tumors. We demonstrated that spatial heterogeneity exists in antibody-binding profiles between stroma-rich and stroma-poor tumor regions. These findings improve our understanding of the complex antibody targeting process and should aid in designing antibodies that show more favorable targeting properties.

## Supplementary information

Supplementary file1
