# Supplementary material for: Modeling the dynamics of antibody–target binding in living tumors
Source: Sci Rep. 2020 Oct 7;10:16764. doi: 10.1038/s41598-020-73711-y (PMC7542163; doi:10.1038/s41598-020-73711-y)
Supplement: Supplementary file 1 — Supplementary file1 [file 41598_2020_73711_MOESM1_ESM.pdf]

Modeling the dynamics of antibody–target binding in living tumors

Yu Tang[1], Yanguang Cao[1,2 #]

[1]Division of Pharmacotherapy and Experimental Therapeutics, UNC Eshelman School of Pharmacy, University of North Carolina at Chapel Hill, Chapel Hill, North Carolina. 27599, United States; [2]Lineberger Comprehensive Cancer Center, School of Medicine, University of North Carolina at Chapel Hill, Chapel Hill, NC 27599, USA

[#] Corresponding author: Yanguang Cao

UNC Eshelman School of Pharmacy, UNC at Chapel Hill.

Tel: (919) 966-4040, Email: yanguang@unc.edu

Conflict of interest: The authors declared no competing interests for this work.

Funding information: This study was supported by National Institute of Health GM119661

Supplementary methods

Heterogeneous binding model (HBM) differential equations

Plasma

$$\frac{d}{dt}A_{plasma} = CL_D \cdot C_{peripheral} - CL_D \cdot C_{plasma} - CL_p \cdot C_{plasma}$$

Other tissues

$$\frac{d}{dt}A_{peripheral} = CL_D \cdot C_{plasma} - CL_D \cdot C_{peripheral}$$

Free cetuximab concentration (C_1) in stroma-poor regions

$$\frac{d}{dt}C_1 = \frac{(1-\sigma_v) \cdot L_p \cdot C_{plasma}}{V_{p\_a}} - \frac{(1-\sigma_L) \cdot L_p \cdot C_1}{V_{p\_a}} - k_{on} \cdot C_1 \cdot R_1 + k_{off\_p} \cdot AR_1$$

Free EGFR concentration (R_1) in stroma-poor regions

$$\frac{d}{dt}R_1 = k_{syn} - k_{deg} \cdot R_1 - k_{on} \cdot C_1 \cdot R_1 + k_{off\_p} \cdot AR_1$$

Cetuximab-EGFR complex concentration (AR_1) in stroma-poor regions

$$\frac{d}{dt}AR_1 = k_{on} \cdot C_1 \cdot R_1 + k_{off\_p} \cdot AR_1 - k_{int} \cdot AR_1$$

Free cetuximab concentration (C_2) in stroma-rich regions

$$\frac{d}{dt}C_2 = \frac{(1-\sigma_v) \cdot L_r \cdot C_{plasma}}{V_{r\_a}} - \frac{(1-\sigma_L) \cdot L_r \cdot C_2}{V_{r\_a}} - k_{on} \cdot C_2 \cdot R_2 + k_{off\_r} \cdot AR_2$$

Free EGFR concentration (R_2) in stroma-rich regions

$$\frac{d}{dt}R_2 = k_{syn} - k_{deg} \cdot R_2 - k_{on} \cdot C_2 \cdot R_2 + k_{off\_r} \cdot AR_2$$

Cetuximab-EGFR complex concentration (AR_2) in stroma-rich regions

$$\frac{d}{dt}AR_2 = k_{on} \cdot C_2 \cdot R_2 + k_{off\_r} \cdot AR_2 - k_{int} \cdot AR_2$$

The RO was calculated by the equation below:

$$\mathrm{RO}(\%) = 100 \cdot \frac{AR_1 \cdot V_{p\_a} + AR_2 \cdot V_{r\_a}}{(AR_1 + R_1) \cdot V_{p\_a} + (AR_2 + R_2) \cdot V_{r\_a} + R0 \cdot (\frac{V_{p\_a}}{f_{av}} + \frac{V_{r\_a}}{f_{av}} - V_{p_a} - V_{r_a})}$$

Sensitivity Analysis

The sensitivity of model to parameters was evaluated by a local sensitivity analysis of the final model and parameters. All of the 8 estimated parameters (k_{deg}, R0, k_{on}, k_{off_p}, k_{off_r}, f_t, TBF, and k_{int}) were included in the sensitivity analysis. Area under *in vivo* RO curve (AUC_{RO}) at three doses (50 mg/kg, 8.5 mg/kg, and 1.0 mg/kg) was chosen as relevant model output and the percentage change in AUC_{RO} was selected as local sensitivity indices. The percentage change in AUC_{RO} with $\pm$ 50% alteration in the model parameters was calculated as below:

$$\%\mathrm{Change} = \frac{AUC_{SIM} - AUC_{\pm 50\%}}{AUC_{SIM}} \cdot 100$$

Where AUC_{SIM} represents the AUC obtained with the optimized parameter set and $AUC_{\pm 50\%}$ refers to the AUC obtained following a 50% increase or decrease in a single parameter value.

Association kinetic assay of the DY605-CTX: NanoLuc-EGFR BRET pair

HEK293 cells stably expressing NanoLuc-EGFR were seeded 24 hours prior to the experimentation at 20,000 cells/well on white 96-well opaque plates and incubated at 37°C/5% CO_2. NanoLuc substrate furimazine was added 5 mins prior to the experiment. DY605-CTX (Dye per antibody ratio [DAR] = 4.6) or DY605-IgG (DAR = 5.6) were added to reach the final concentrations at 1, 3, 6, 12, and 25 nM. The data collection and quantification were described in our previous study[1]. The BRET ratios were calculated every 4 mins for up to 25 mins.

Supplementary Figure 1

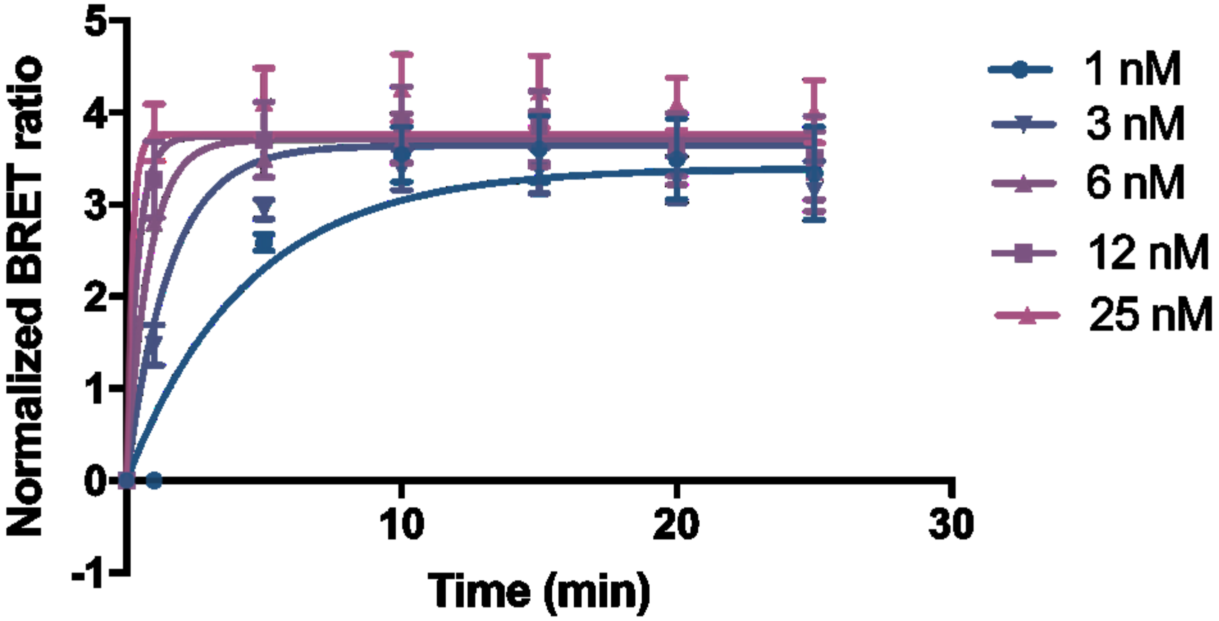

Figure S1. The association kinetics of the DY605-CTX:NanoLuc-EGFR BRET pair. BRET ratios were baseline-corrected to the negative controls (DY605-IgG). Data are shown as mean ± SD. Curves were fitted to the association kinetic model with multiple radioligand concentrations. K_{on} = 0.2 $nM^{-1}min^{-1}$, k_{off} = 0.02 min^{-1}, and K_D = 0.10 nM.

Supplementary Figure 2

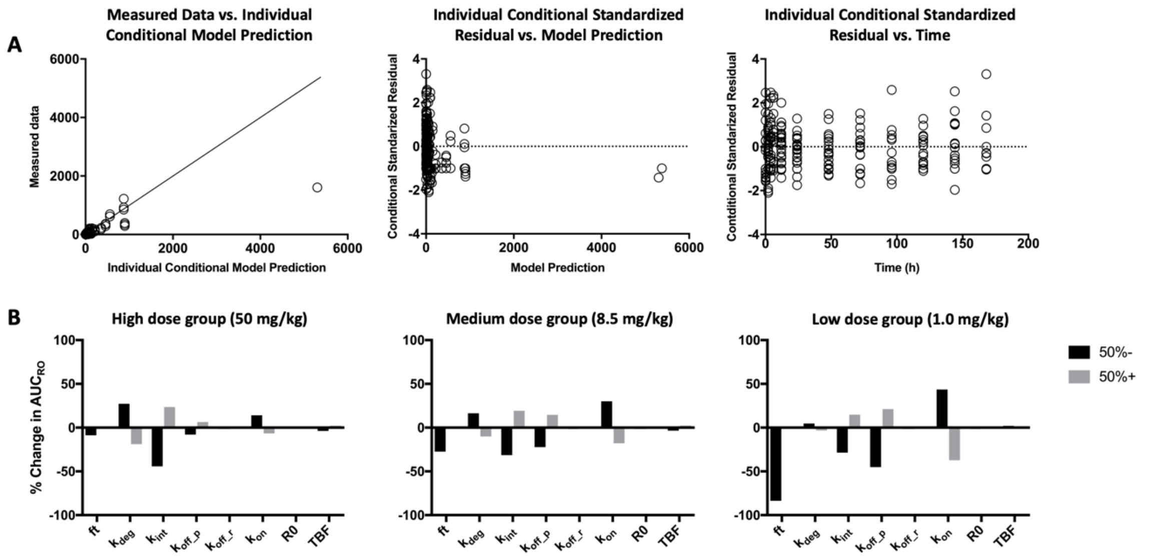

Figure S2. The model diagnostic plots and sensitivity analysis. (A) The measured data vs. individual conditional model prediction plots, the individual conditional standardized residual vs. model prediction plot, and the individual conditional standardized residual vs. time plot. **(B)** The local sensitivity analysis of the HBM. k_{deg}: EGFR degradation rate; R0: EGFR initial concentration in tumor stroma-rich and stroma-poor space; k_{on}: cetuximab-EGFR association rate; k_{off_p}: cetuximab-EGFR dissociation rate in stroma-poor regions; k_{off_r}: cetuximab-EGFR dissociation rate in stroma-rich regions; f_t: ratio of tumor stroma-poor volume over total space; TBF: tumor blood flow per 1L tumor; k_{int}: cetuximab-EGFR internalization rate.

Supplementary Figure 3

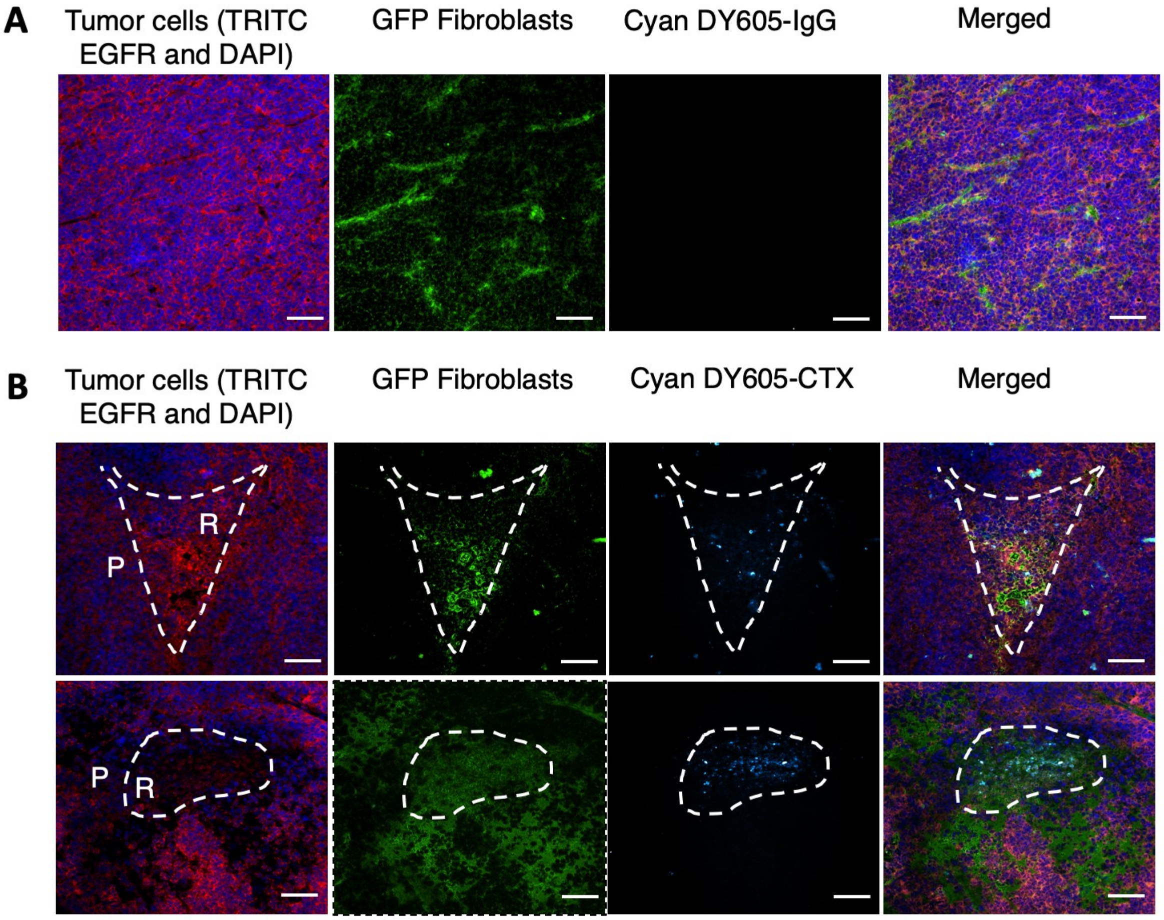

Figure S3. Representative immunofluorescence (IF) images showing the histology of the tumor collected at the end of the bioluminescence resonance energy transfer (BRET) imaging study. (A) The Cyan signals denoted bound DY605-labeled cetuximab rather than non-specific binding. No residual antibodies were observed for nonspecific IgG, suggesting that the residual antibodies were associated with Fab binding and not with non-specific binding. **(B)** The spatial distribution of the antibody (Cyan DY605-CTX), tumor-associated fibroblast (GFP Fibroblasts), and EGFR-positive tumor cells (TRITC EGFR and DAPI). Area P represents the tumor area without many stroma cells and with evenly distributed tumor cells. Area R represents the stroma-rich area, where tumor cells were surrounded by tumor-associated fibroblasts. Cyan signal denoted the total antibodies, including free DY605-CTX and bound DY605-CTX. Scale bar = 100 μm.

Supplementary Table 1

Table S1 Heterogeneous distribution model (HDM) parameter estimations

Parameter	Unit	Definition	Estimation (CV%)
k_{deg}	h^{-1}	EGFR degradation rate	0.00082 (101%)
R0	nM	EGFR initial concentration in tumor stroma-rich and stroma-poor areas	0.0027 (292%)
k_{on}	$nM^{-1}\cdot h^{-1}$	Cetuximab-EGFR apparent association rate	0.76 (54%)
k_{off}	h^{-1}	Cetuximab-EGFR apparent dissociation rate	0.57 (20%)
f_t		Ratio of tumor stroma-poor space volume over total space	0.55 (6.0%)
TBF_p	h^{-1}	Tumor Blood Flow per 1L tumor at stroma-poor regions	1.02 (37%)
TBF_r	h^{-1}	Tumor Blood Flow per 1L tumor at stroma-rich regions	0.0067 (39%)
k_{int}	h^{-1}	Cetuximab-EGFR internalization rate	0.04 (13%)

Reference

1 Tang, Y., Parag-Sharma, K., Amelio, A. L. & Cao, Y. A Bioluminescence Resonance Energy Transfer-Based Approach for Determining Antibody-Receptor Occupancy In Vivo. *iScience* **15**, 439-451, doi:10.1016/j.isci.2019.05.003 (2019).
